# Methoprene application and diet protein supplementation to male melon fly, *Bactrocera cucurbitae*, modifies female remating behavior

**DOI:** 10.1111/1744-7917.12073

**Published:** 2013-12-28

**Authors:** Ihsan ul Haq, Marc J B Vreysen, P E A Teal, Jorge Hendrichs

**Affiliations:** 1 Insect Pest Control Laboratory, Joint, FAO/IAEA Agriculture and Biotechnology Laboratories A-2444Seibersdorf, Austria; 2 National Agricultural Research CentrePark Road, Islamabad, 45500, Pakistan; 3 Center for Medical, Agricultural and Veterinary Entomology, USDA, ARSGainesville, Florida, 32604, USA; 4 Insect Pest Control Section, Joint, FAO/IAEA DivisionIAEA A-1400, Vienna, Austria

**Keywords:** *Bactrocera cucurbitae*, methoprene, protein rich diet, remating behavior, SIT, Tephritidae

## Abstract

Methoprene (an analogue of juvenile hormone) application and feeding on a protein diet is known to enhance male melon fly, *Bactrocera cucurbitae* Coquillett (Diptera: Tephritidae), mating success. In this study, we investigated the effect of these treatments on male *B. cucurbitae*'s ability to inhibit female remating. While 14-d-old females were fed on protein diet, 6-d-old males were exposed to one of the following treatments: (i) topical application of methoprene and fed on a protein diet; (ii) no methoprene but fed on a protein diet; (iii) methoprene and sugar-fed only; and (iv) sugar-fed, 14-d-old males acted as controls. Treatments had no effect on a male's ability to depress the female remating receptivity in comparison to the control. Females mated with protein-deprived males showed higher remating receptivity than females first mated with protein-fed males. Methoprene and protein diet interaction had a positive effect on male mating success during the first and second mating of females. Significantly more females first mated with sugar-fed males remated with protein-fed males and females first mated with methoprene treated and protein-fed males were more likely to remate with similarly treated males. Females mating latency (time to start mating) was significantly shorter with protein-fed males, and mating duration was significantly longer with protein-fed males compared with protein-deprived males. These results are discussed in the context of methoprene and/or dietary protein as prerelease treatment of sterile males in area-wide control of melon fly integrating the sterile insect technique (SIT).

## Introduction

Female insects show a wide range of mating strategies and physiological responses to mating. In some species, the female mates only once, females of other species remain sexually unreceptive for a period after mating but then remate, while still others show no decrease in receptivity after each mating (Thornhill & Alcock, 1983). Females may benefit by multiple mating, especially with different males (i.e., polyandry) (Andersson, 1994). These benefits may be direct through increased fertility or receipt of nuptial gifts (Thornhill & Alcock, 1983; Vahed, 1998), or may be indirect through increased offspring fitness (Jennions & Petrie, 2000).

Indirect, or genetic benefits of multiple mating are only available to polyandrous females (Arnqvist & Nilsson, 2000). Polyandry imposes a fitness cost to a male which mates with a female that subsequently remates, and the first male's sperm's likelihood of fertilizing the female's eggs decreases, especially in species with last-male sperm precedence. This will therefore select for traits in males that prevent females from remating, or which increase the probability of the female using his sperm quickly by inducing oviposition. Females that resist these male traits and choose to get benefits from multiple mating which can lead to an evolutionary arms race between the sexes for traits that control remating and oviposition (Parker, 1979; Pitnick *et al*., 2001). Alternatively, while it is disadvantageous to the first male mating with a female for her to remate, it is also clearly advantageous to a second male to mate with this female. This leads to a selective paradox in which male traits should be selected which first stimulate a female to mate or remate with him, but which then stop that female remating after his mating. There are thus recognized to be both offensive male mating traits, for example, pheromone calling, and defensive male mating traits, for example, reducing female receptivity by which the male is “defending” his ejaculate (Friberg *et al*., 2005). Understanding these processes is essential and highly relevant for increasing the effectiveness of the sterile insect technique (SIT). SIT is a pest control strategy whereby sterilized male flies are reared and mass released for the purpose of mating with wild females by outcompeting fertile wild males, so controlling or stopping reproduction of the wild pest population (Knipling, 1955). This technique has been widely used around the world, especially against dipterous pests such as tsetse fly, screwworm, and fruit flies (Vreysen *et al*., 2000; Wyss, 2000; Koyama *et al*., 2004).

The melon fly, *Bactrocera cucurbitae* (Coquillett) (Diptera: Tephritidae), is an economically important pest of fruits and vegetables. This species causes severe economic losses, both directly by damaging cucurbit fruits and vegetables (White & Elson-Harris, 1992) and indirectly by interfering the trade of fresh fruits and vegetables due to its status of quarantine pest insect (Kakinohana, 1994). Relying on traditional chemicals for the control of tephritid pests entails increasing environmental concerns (Rössler, 1989). SIT, applied as a component of an area-wide integrated pest management, is a well-established environmentally friendly technique used for melon fly suppression (Vargas *et al*., 2004; Jang *et al*., 2008) or eradication (Koyama *et al*., 2004). For the success of sterile insect release programmes, the mass-reared sterile males should be able to survive in the field, compete with wild males for mating, successfully transfer sterile sperm to wild females and inhibit female remating (Hendrichs *et al*., 2002). Long-term mass-rearing is known to have adverse effects on male mating competitiveness (Iwahashi *et al*., 1983). Mass-rearing environment also decrease the sterile male's ability to decrease wild female remating that ultimately can undermine the success of sterile insect release programmes (Kuba & Itô, 1993; Nitzan *et al*., 1993; Vera *et al*., 2003).

In tephritids diet supplementation increases the male's ability to depress the female remating receptivity. For example, *Anastrepha ludens* (Loew) and *Anastrepha obliqua* (Macquart) males fed on protein diet induced longer refractory periods in females than males fed only sugar (Aluja *et al*., 2009). Similarly, laboratory studies on *Ceratitis capitata* (Wiedemann) showed that females mated with protein-deprived males had higher remating receptivity than females first mated with protein-fed males (Blay & Yuval, 1997; Gavriel *et al*., 2009). In both *C. capitata* and *Bactrocera tryoni* (Froggatt), it has been demonstrated that female remating behavior is modulated by male accessory gland fluids, which are modified by male's feeding on protein diet (Jang, 1995; Radhakrishnan & Taylor, 2007, 2008). Kuba and Itô (1993) suggested that male accessory glands substances may also depress female remating in the polyandrous *B. cucurbitae*. In addition to accessory glands fluid, increased mating duration has a significant negative effect on *B. cucurbitae* female remating receptivity, although sperm storage *per se* has no effect on female remating inhibition (Yamagishi & Tsubaki, 1990; Kuba & Itô, 1993).

In insects, the development of male accessory glands and their secretions are regulated by juvenile hormone (JH) and application of JH analogues can also regulate accessory glands development and secretions (Gillott & Gaines, 1992; Braun & Wyatt, 1995; Parthasarathy *et al*., 2009). Application of methoprene (a JH analogue) and a protein diet enhanced *B. cucurbitae* male mating competitiveness and mating duration (Haq *et al*., 2010). We hypothesized, these treatments, may also modulate female’ remating receptivity. In this study we tested this hypothesis by assessing: (i) whether the male condition (methoprene application and protein diet) affects the probability that a virgin female copulated with will remate; and (ii) whether the mate choice of females that do remate is affected by the condition of their first sexual partner.

## Materials and methods

### Strain and rearing

A genetic sexing strain of the melon fly, developed by ARS-USDA, Hawaii (McInnis *et al*., 2004), was used for all experiments. The genetic sexing strain of *B. cucurbitae* is based on pupal colour, with male pupae being brown (wild-type) from female pupae white (mutant) (McInnis *et al*., 2004). The colony was maintained on wheat-based, modified standard Seibersdorf diet (Hooper, 1987) at the FAO/IAEA Agriculture and Biotechnology Laboratories, Seibersdorf, Austria. Following emergence, the flies were sexed and maintained in a fully internal, controlled environment room (24 ± 1 °C, 60% ± 5% RH, 14 L : 10 D photoperiod).

### Treatments

There were 4 treatments of adult males:

- Topical application of the juvenile hormone analogue, methoprene (M) and access to sugar and hydrolyzed yeast (protein diet [P]) as adult food (M+P+).
- No methoprene application but access to sugar and hydrolyzed yeast as adult food (M−P+).
- Topical application of methoprene and only sugar as adult food (M+P−).
- No methoprene application and only sugar as adult food; “control” male (M−P−).

Methoprene was applied topically 3–4 h after adult emergence at a rate of 5 μg in 1 μL acetone solution per male by immobilizing males in a net bag (FAO/IAEA/USDA, 2003) and applying the solution via a pipette through the net onto the dorsal surface of the thorax. Previous work has shown that application of only acetone has no effect on male mating success (Teal *et al*., 2000), hence acetone was not included as a control treatment. Males from each treatment were maintained in separate 30-cm high × 20-cm diameter cylindrical screen cages containing the type of food assigned for each treatment. In treatments without protein feeding (P−) only water and sugar were supplied to the flies *ad libitum*. In the treatments with protein (P+), hydrolyzed yeast was added to the sugar in a proportion of 3 : 1, sugar : hydrolyzed yeast, and supplied with water *ad libitum*. Virgin females used in the experiments were maintained in 30-cm × 20-cm diameter cylindrical screen cages without exposure to males; they were provided with the 3 : 1 sugar : hydrolyzed yeast diet and water *ad libitum*.

Topical methoprene application accelerates sexual maturity in protein-fed males, with these males sexually mature at 5–7 d, while sugar-fed males reach sexual maturity at 14 d (Haq *et al*., 2010). Therefore, 6-d-old treated M+P+, M−P+ and M+P− males and 14-d-old M−P− (control) males were used in experimental treatments.

### Experiment 1. Ability of males to inhibit female remating in field cage test

The experiment was conducted in screened field cages (4 m^2^ base and 1.8 m in height), containing a single potted *Citrus sinensis* (Osbeck), a nonhost tree (height 1.7 m with a canopy of about 1.5 m in diameter). The plant provided an architecturally complex substrate upon which mating could occur. Melon fly males form mating aggregations on nonhost plants at the edge of melon fields and matings occur at late dusk (Iwahashi & Majima, 1986), when the trials were run. A temperature of 25 ± 2 °C and 65% ± 5% RH was maintained throughout mating trials. Twenty males of each of the following treatments, M+P+, M−P+, M+P−, and M−P−, were released into the cage 90 min before sunset. Males from different treatments had been marked on the thorax by different colors of water-based paint 1 d earlier. Fifteen minutes later, 20 sexually mature females (14-d old) were released and mating success under the 4 : 1 male to female sex ratio was observed until pair-formation ceased due to darkness. The experiment was repeated 8 times and different flies were used in each replication.

The mating couples were collected separately in plastic vials and allowed to complete mating overnight. The next morning, now uncoupled females were removed, marked with the color of their male partner on their thorax, and supplied with sugar and water for 1 d. Forty-eight hours after first mating, all mated females (in the same cage as they were during the first mating) were exposed again to equal numbers of virgin males as above and mating behavior again observed and recorded.

### Experiment 2. Field cage pairwise evaluation of male ability to inhibit the female remating

In the previous experiment M+P− males were found to be not competitive against males of other treatments and were subsequently excluded from this second experiment. Experiment 2 was designed to evaluate the ability of males fed a protein diet, with and without methoprene application, to inhibit female remating as compared to M−P− males. The experimental setup was split into 2 pairwise combinations: (i) males M+P+ (6-d old) were tested against M−P− males (14-d old); and (ii) males M−P+ (6-d old) were tested against M−P− males (14-d old). Feeding and methoprene treatment were the same as previously described, as were the marking procedures. Both combinations of males were rotated among field cages during replications to avoid confounding effects of environmental conditions of particular field cages on mating behavior. The experiment was replicated 6 times (2 replicates for each combination simultaneously per day). Within each field cage, 40 males of each treatment were released 90 min before sunset and 15 min later 40 females (14-d age) were released. Mating couples were collected separately in plastic vials as soon as they coupled and left together overnight. The next morning, females were marked on the thorax with the same color as their mate and maintained on sugar and water for 1 d. Forty-eight hours after the first mating, females were exposed again to equal numbers of either virgin M+P+ males (6-d old) and M−P− males (14-d old) or M−P+ males (6-d old) and M−P− males (14-d old). The number of males from each treatment was equal to the total number of mated females, giving a 2 : 1 male to female ratio. Second mating of females was evaluated using the same methodology as the first mating. Six replications were carried out.

### Experiment 3. Remating ability of females under laboratory conditions

Laboratory experiments were conducted in the controlled environment room as described above, except that the period of darkness started in the morning (to facilitate the running of trials). The experiment was conducted in small cages (Plexiglass 5 × 5 × 10 cm, 1 side screened and a rubber stopper to allow insertion of the aspirator) and consisted of 2 pairwise male combinations: (i) males M+P+ (6-d old) against males M−P− (14-d old), and (ii) males M−P+ (6-d old) against males M−P− (14-d old), and (iii) a noncompetitive arena involving only M−P− males (14-d old). The noncompetitive mating trial was run as the numbers of females mating with M−P− males in competition with M+P+ or M−P+ males were not sufficient to assess their second mating, so this third trial was run to obtain females for the second mating exposure.

For each small cage trial, 2 virgin males (1 of each treatment) were released into a cage 90 min before onset of darkness, and then 1 virgin female was released into the cage before the onset of darkness. A total of 150 cages were used during each replication (50 cages for each combination) and 4 replications were run. Pair formation was observed under semi-dark conditions (lux > 50). The nonmated males were removed from the cages as soon as mating occurred and couples were allowed to complete mating. Mating latency (time to start mating) and mating duration of the first mating was recorded.

The next day, now uncoupled females were placed individually into different small cages and supplied for 1 d with sugar and water. Forty-eight hours after the first mating the females were exposed to virgin males for a second mating. Only females that had copulated for more than 180 min were selected for the second mating trial. The experimental procedure was the same as in the first mating. Females mated with M+P+ males in the first mating were divided into 3 groups. One group of these females was allowed to mate with M+P+ or M−P+ males, a second group with M+P+ or M−P− males, and a third group with M−P+ or M−P− males. Thus, each female was given the opportunity to mate with 2 males, one each of the treatment categories described. The age of the males was similar to that of the first exposure. Similarly, females mated with M−P+ in the first mating, or with M−P− in the first mating, were also divided into 3 groups and each group had the same combination of males and sex ratio. Mating latency and mating duration in the second mating were also observed and recorded. The remating experiment was replicated 4 times, and each replication used 62–68 cages for each pairwise treatment.

### Data analysis

The effect of methoprene, protein diet, and their interaction to mate with female and their influence on female remating was assessed by binary logistic regression where 1 was mated and 0 was unmated. The effect of blocking and replications when nonsignificant were removed from the model to increase the power of analysis. The likelihood of female mating and remating with either of the treated males tested in Experiment 2 were assessed by chi-square test. Logistic ANOVA was used to differentiate the differences in female remating with differently treated males among 3 groups in Experiment 3 and complementary pairwise comparisons (Scheffé's Test; suitable for unequal sample size) were performed (Zar, 1996). Time to start mating (referred to as latency) was calculated as the time elapsed from the release of females until initiation of a given mating. Mating duration was calculated as the time when mating pairs separated (no longer in genital contact) minus the time at which they started to mate. Differences in mating latency and mating duration data having normal distribution were analyzed by one-way ANOVA using a General Linear Model and complementary pairwise comparisons of means (Scheffé's Test) were performed. The significance value used in tests was 95% (α = 0.05). Data were analyzed by using Statistica software (StatSoft, 2000).

## Results

### Experiment 1. Ability of males to inhibit female remating in field cage test

**Fig. 1:**
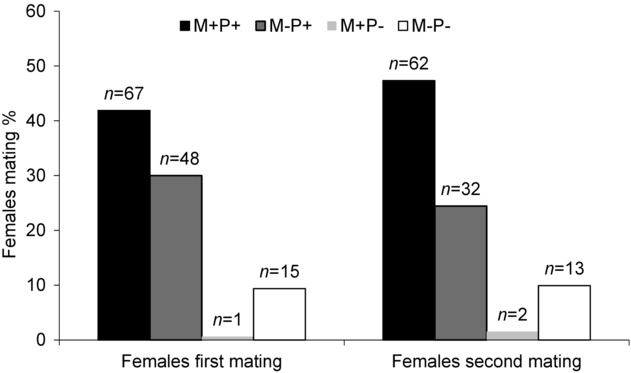
Cumulative mating percentage of *Bactrocera cucurbitae* females in their first and second mating. Females were exposed simultaneously to virgin M+P+, M−P+, M+P− and M−P− males during first and second mating (48 h after 1st mating) in field cages. Males were 6-d-old treated either with methoprene and protein (M+P+), no methoprene and protein (M−P+), methoprene and only sugar (M+P−), or 14-d-old treated with no methoprene and only sugar (M−P−).

Out of 160 females evaluated, 131 females were mated during the first mating opportunity (Fig. 1). Application of methoprene (M+) had no effect on male mating success (*G* = 0.24, df = 1, *P* = 0.62), but access to a protein diet (P+) increased male mating success (*G* = 108.09, df = 1, *P* < 0.01) and the interaction of methoprene and protein (M+P+) also had significantly enhanced male mating success (*G* = 6.07, df = 1, *P* < 0.01). None of the treatments had any effect on female remating receptivity (*G* = 3.6, df = 1, *P* = 0.057) and 84% of females, 109 of 130 tested for second mating, were remated. During the second mating of females, the effect of methoprene and protein supplementation on male mating success was similar to effects during first mating of females. Methoprene only had no effect but access to protein diet (*G* = 10.13, df = 1, *P* < 0.01) and interaction of methoprene application with protein diet had a significant positive effect (*G* = 6.29, df = 1, *P* < 0.01) on male mating success during the second mating of female (Fig. 1).

### Experiment 2. Field cage pairwise evaluation of male ability to inhibit female remating

**Fig. 2:**
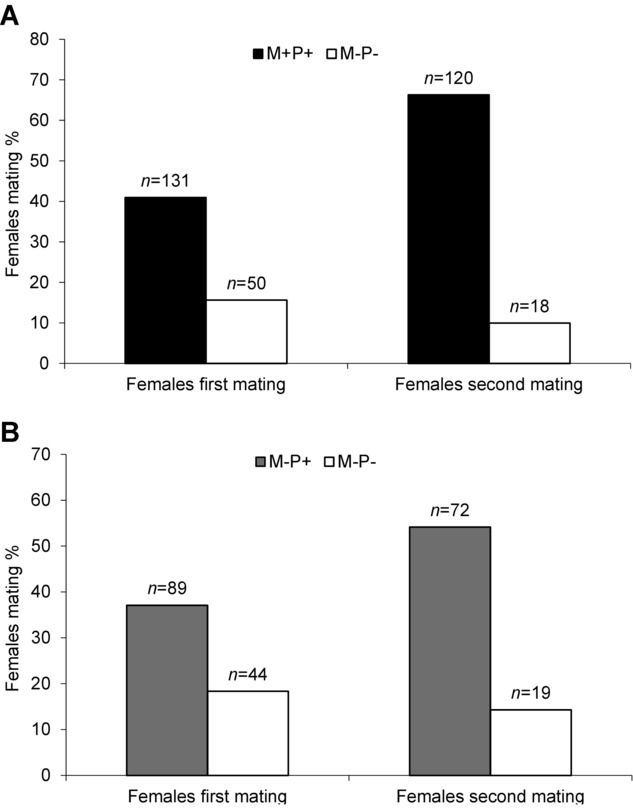
Mating percentage of *Bactrocera cucurbitae* females in their first and second mating. Females were exposed to (A) virgin (M+P+ and M−P−) or (B) (M−P+ and M−P−) males separately during their first and second mating (48 h after 1st mating) in field cages. Males were 6-d-old treated with either methoprene and protein (M+P+), no methoprene and protein (M−P+), methoprene and only sugar (M+P−), or 14-d-old treated with no methoprene and only sugar (M−P−).

Females were more likely to mate with M+P+ than M−P− (χ^2^ = 37.43, *P* < 0.01) and M−P+ than M−P− males (χ^2^ = 18.54, *P* < 0.05) in their separate pairwise comparisons during the first mating. None of the male treatments depressed female remating receptivity. However, females first mated with M−P− males were significantly more likely to remate with M+P+ (χ^2^ = 77.02, *P* < 0.01) or M−P+ males (χ^2^ = 34.69, *P* < 0.001) as compared to M−P− males separately (Fig. 2).

### Experiment 3. Remating ability of females under laboratory conditions

**Fig. 3:**
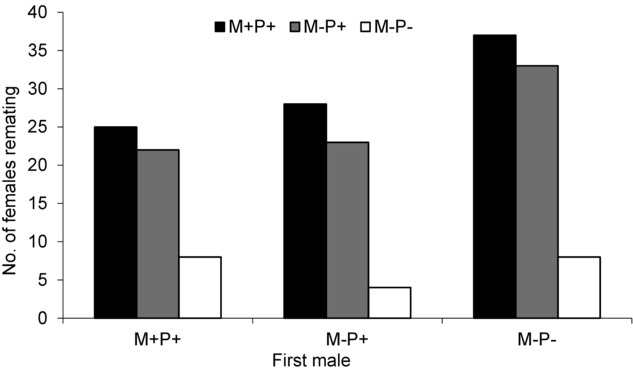
Cumulative number of females *Bactrocera cucurbitae* remating with M+P+, M−P+, or M−P− males in single pair cages in the laboratory. The females first mated with M+P+, M−P+, and M−P− males were exposed 48 h after first mating to 1 of 3 combinations of virgin males, either M+P+ versus M−P+, M+P+ versus M−P−, or M−P+ versus M−P−. Males were 6-d-old treated with either methoprene and protein (M+P+), no methoprene and protein (M−P+), methoprene and only sugar (M+P−), or 14-d-old treated with no methoprene and only sugar (M−P−).

Females showed significantly different remating tendencies according to the treatment their first mate had experienced (*F*_2,253_ = 10.85, *P* < 0.01). Females mated first with M+P+ or M−P+ males showed similar remating receptivity (Scheffé's test, *P* = 0.78), but females mated first with M−P− males showed significantly higher remating receptivity as compared to females mated first (Scheffé's test, *P* < 0.001) with M+P+ or M−P− males (Fig. 3).

A comparison of effect of first male treatment on female remating tendency within each group of males showed that females mated first with M+P+ males mated significantly more with M+P+ males as compared to M−P+ males (within the group M+P+ vs. M−P+) and significantly less with sugar-fed (M−P−) males (within the group M+P+ vs. M−P− and M−P+ vs. M−P−). Females mated first with M−P+ and M−P− males had a similar remating tendency with M+P+ and M−P+ males, but remated significantly more with P+ males within the two other groups (Table 1).

**Table 1:** The number of female *Bactrocera cucurbitae* remating exposed 48 h after first mating to 1 of 3 combinations of males†, either M+P+ versus M−P+, M+P+ versus M−P−, or M−P+ versus M−P− in single pair cages in the laboratory. The significant differences in female remating by male treatment within each group of males (rows) are represented by (*) (Logistic regression, *P* < 0.05). The number of females tested for remating is shown in parenthesis

	No. of female remated by differently treated male within each male group					
	
Female 1st mating by male treatment	M+P+ versus M−P+		M+P+ versus M−P−		M−P+ versus M−P−	
			
	M+P+	M−P+	M+P+	M−P−	M−P+	M−P−
M+P+	15* (28)	8 (28)	10* (31)	4 (31)	14* (29)	4 (29)
M−P+	15 (31)	13 (31)	13* (29)	1 (29)	10* (25)	3 (25)
M−P−	16 (29)	12 (29)	21* (28)	4 (28)	21* (29)	4 (29)

† Males were 6-d-old treated either with methoprene and protein (M+P+), no methoprene and protein (M−P+), methoprene and only sugar (M+P−), or 14-d-old treated with no methoprene and only sugar (M−P−).

### Mating latency and mating duration

The mating latency of virgin females to start their first mating was shortest for matings with M+P+ males, significantly longest for matings with M−P− males (Scheffé's test, *P* = 0.03), and not different from these 2 male types for matings with M−P+ males (Scheffé's test, *P* = 0.14). The females showed shorter but similar mating latency with M+P+ and M−P+ males compared with M−P− males (Scheffé's, *P*<0.01) during their second mating.

In terms of mating duration during their first mating, females had similar mating duration with M+P+ and M−P+ males, but significantly shorter matings with sugar-fed (M−P−) males (Scheffé's test, *P* < 0.01). Similar to the first mating, females had similar mating duration with M+P+ and M−P+ males but significantly longer as compared to mating with M−P− males (Scheffé's test, *P* < 0.01) during their second mating (Table 2).

**Table 2:** Mating latency (time to the start of mating) (mean ± SE) and mating duration (mean ± SE) by *Bactrocera cucurbitae* females during first and second mating (48 h after 1st mating). The females first mated with M+P+, M−P+ and M−P− males† were re-exposed to virgin males in groups such as M+P+ versus M−P+, M+P+ versus M−P−, and M−P+ versus M−P− in single pair cages in the laboratory during their second mating. Within each row mean values with different letters are significantly different (Scheffé's test, *P*< 0.05)

Female mating latency (min) by male treatment						Female mating duration (min) by male treatment					
	
Mating latency in 1st mating			Mating latency in 2nd mating			Mating duration in 1st mating			Mating duration in 2nd mating		
			
M+P+	M−P+	M−P−	M+P+	M−P+	M−P−	M+P+	M−P+	M−P−	M+P+	M−P+	M−P−
33 a	43 ab	68 b	32 a	39 a	67 b	398 a	396 a	192 b	427 a	428 a	243 b
(6.36)	(10.24)	(8.02)	(6.16)	(8.44)	(10.85)	(23.68)	(25.11)	(16.11)	(20.62)	(30.92)	(38.30)

† Males were 6-d-old treated either with methoprene and protein (M+P+), no methoprene and protein (M−P+), methoprene and only sugar (M+P−), or 14-d-old treated with no methoprene and only sugar (M−P−).

## Discussion

The results showed that females of the *B. cucurbitae* genetic sexing strain had a high remating tendency, ranging between 50% and 90%. These results contrast with estimates by Kuba and Itô (1993) who reported 35%–45% females remating 48 h after the first mating in a nongenetic sexing strain originated from Japan.

Female remating was higher in those field cage experiments that had a higher density of flies. Fly density and male to female sex are reported to have effect on Mediterranean fruit fly, *C. capitata* female remating receptivity (Vera *et al*., 2002; Mossinson & Yuval, 2003; Kraaijeveld *et al*., 2005).

A protein diet enhanced male mating success during the female's first and second mating. But protein diet did not depress female's remating receptivity in field cage experiments. However, in laboratory experiments, females that mated first with protein-deprived males showed a higher remating receptivity than females mated first with protein-fed males. A similar effect of protein diet has been reported in *C. capitata* and several species of *Anastrepha* where females mated first with protein deprived males had a shorter refractory period under laboratory conditions (Blay & Yuval, 1997; Aluja *et al*., 2009).

Application of methoprene only (a juvenile hormone analogue) has no effect on male mating success or ability to inhibit the female remating but interaction of methoprene application and protein diet had a significant effect to enhance the male mating success compared with protein-fed or protein-deprived males during the female's first and second mating. However, this treatment had no effect on female remating receptivity.

The mechanism by which males depress the female *B. cucurbitae* remating receptivity is not fully understood. The presence or absence of sperm in the spermathecae appears to have no effect on *B. cucurbitae* female remating, while mating duration in the first mating affects the female remating receptivity (Kuba & Soemori, 1988; Kuba & Itô, 1993). Females copulating for less than 3 h, are reported to remate within 2 d after their first mating but females copulating for more than 10 h remated after 12th day of their first mating (Kuba & Soemori, 1988). Nevertheless the observations in the laboratory experiment showed that mean mating duration by protein-fed males was more than 7 h but we did not find that longer mating duration reduced remating receptivity. The difference between our results and those of previous studies may be due to the different strains: we tested a strain originating in Hawaii and previous studies tested wild or laboratory-adapted flies from Japan.

Kuba and Itô (1993) suggested that accessory gland substances may also affect remating receptivity. Application of the juvenile hormone (JH) analogue regulates male accessory glands development and secretion (Parthasarathy *et al*., 2009). Thus, the assumption that application of JH analogue (methoprene) modulates male ability to suppress female remating was not supported by these results. This may be because laboratory colonized females were less sensitive to male accessory gland substances as suggested by Kuba and Itô (1993) also. However, females mated first with methoprene treated and protein-fed males were more likely to remate with methoprene treated and protein-fed males than all other treated males and this trend was uniform in all experiments.

Haq *et al*. (2010) reported that *B. cucurbitae* males treated with methoprene and protein-fed had the shortest mating latency than nontreated but protein-fed or protein-deprived males under field cage conditions. However, no additive effect of methoprene application on mating latency was observed in this study but protein diet did have an effect. The discrepancy between these 2 studies may due to differences in experimental conditions. The current results are from smaller cages under laboratory conditions where the effect of methoprene treatment was not as visible as under field cage conditions.

For effective SIT application, independently of the number of wild female rematings, mass-reared sterile males should reduce wild female remating to the same degree as by wild males (Whitten & Mahon, 2005). Even though methoprene treated and protein-fed males did not provide additional inhibition of female remating, but these treatments have certain other advantages like accelerated male's sexual maturity and enhanced mating competitiveness (Haq *et al*., 2010). Accelerated male sexual maturity can reduce the cost of holding sterile males in release facilities and enhanced mating competitiveness may add to the success of SIT application. Furthermore, the fact that female mated and remated preferentially with protein-fed males is encouraging for improved SIT application.

In this study on a genetic sexing strain, a higher female remating receptivity was observed compared to results from nongenetic sexing strain (Kuba & Itô, 1993) and male treatment had no effect on males ability to suppress female remating. Kuba and Itô (1993) reported that mass-rearing had also an effect to increase the female remating receptivity. Thus, higher remating receptivity of genetic sexing strain females may be due to the effect of laboratory colonization or a strain specific character. To answer these questions we suggest that comparative studies on remating behavior of *B. cucurbitae* wild, laboratory adopted, and genetic sexing strain should be carried out.
